# Look me in the eye: evaluating the accuracy of smartphone-based eye tracking for potential application in autism spectrum disorder research

**DOI:** 10.1186/s12938-019-0670-1

**Published:** 2019-05-03

**Authors:** Maximilian A. R. Strobl, Florian Lipsmeier, Liliana R. Demenescu, Christian Gossens, Michael Lindemann, Maarten De Vos

**Affiliations:** 1 Wolfson Centre for Mathematical Biology, Mathematical Institute, University of Oxford, Radcliffe Observatory Quarter, OX2 6GG Oxford, UK; 2 Department of Integrated Mathematical Oncology, Moffitt Cancer Center, Magnolia Drive, 12902 Tampa, USA; 3 Roche Pharma Research and Early Development, pRED Informatics, Roche Innovation Center, F. Hoffmann-La Roche Ltd, Basel, Switzerland; 4 Department of Engineering Science, Institute of Biomedical Engineering, University of Oxford, Old Road Campus Research Building, OX3 7DQ Oxford, UK

**Keywords:** Gaze tracking, Mental disorders, m-Health, Biomedical monitoring

## Abstract

**Background:**

Avoidance to look others in the eye is a characteristic symptom of Autism Spectrum Disorders (ASD), and it has been hypothesised that quantitative monitoring of gaze patterns could be useful to objectively evaluate treatments. However, tools to measure gaze behaviour on a regular basis at a manageable cost are missing. In this paper, we investigated whether a smartphone-based tool could address this problem. Specifically, we assessed the accuracy with which the phone-based, state-of-the-art eye-tracking algorithm iTracker can distinguish between gaze towards the eyes and the mouth of a face displayed on the smartphone screen. This might allow mobile, longitudinal monitoring of gaze aversion behaviour in ASD patients in the future.

**Results:**

We simulated a smartphone application in which subjects were shown an image on the screen and their gaze was analysed using iTracker. We evaluated the accuracy of our set-up across three tasks in a cohort of 17 healthy volunteers. In the first two tasks, subjects were shown different-sized images of a face and asked to alternate their gaze focus between the eyes and the mouth. In the last task, participants were asked to trace out a circle on the screen with their eyes. We confirm that iTracker can recapitulate the true gaze patterns, and capture relative position of gaze correctly, even on a different phone system to what it was trained on. Subject-specific bias can be corrected using an error model informed from the calibration data. We compare two calibration methods and observe that a linear model performs better than a previously proposed support vector regression-based method.

**Conclusions:**

Under controlled conditions it is possible to reliably distinguish between gaze towards the eyes and the mouth with a smartphone-based set-up. However, future research will be required to improve the robustness of the system to roll angle of the phone and distance between the user and the screen to allow deployment in a home setting. We conclude that a smartphone-based gaze-monitoring tool provides promising opportunities for more quantitative monitoring of ASD.

**Electronic supplementary material:**

The online version of this article (10.1186/s12938-019-0670-1) contains supplementary material, which is available to authorized users.

## Background

Autism spectrum disorders (ASD) describes a set of developmental disabilities characterised by “deficits in social communication and social interaction” [1]. As the name “spectrum” suggests, the nature of the impairments and their severities vary from person to person, with some subjects being able to complete university degrees and live independent lives, whereas others need life-long assistance with daily living [2]. Despite this variability there are certain commonalities: Already in one of the earliest accounts of autism, the author noted that the subject “never looked into anyone’s face” [3]. A series of eye-tracking studies since then have established that, when shown the image of a face, ASD patients spend less time fixating on the eyes and more time exploring the mouth or objects in the surroundings (see [4] for an extensive review). In fact, gaze abnormalities are one of the criteria used to diagnose ASD [1].

While abnormal gaze behaviour is not the cause for the difficulties which subjects with ASD experience, it has been proposed that it might provide a quantifiable feature for monitoring the condition over time, and to evaluate the efficacy of treatments [4, 5]. However, a key challenge that has prevented further exploration of this idea so far has been the lack of access to a suitable eye-tracking device. Traditional eye-tracking devices are inconvenient for widespread home use, as they are costly, often not very portable, and require expertise to set up and run [6, 7]. But thanks to recent advances new solutions are emerging that allow eye-tracking using only the camera of a laptop or smartphone. These approaches use machine-learning techniques, such as support vector methods [8], Gaussian processes [9], or neural networks, [10, 11], and hold great potential for longitudinal gaze behaviour monitoring in ASD patients. We envision the development of a mobile-monitoring tool in which patients regularly perform a series of tests on their smartphone that measure their gaze behaviour and so provide insights into the development of their condition over time.

**Fig. 1:**
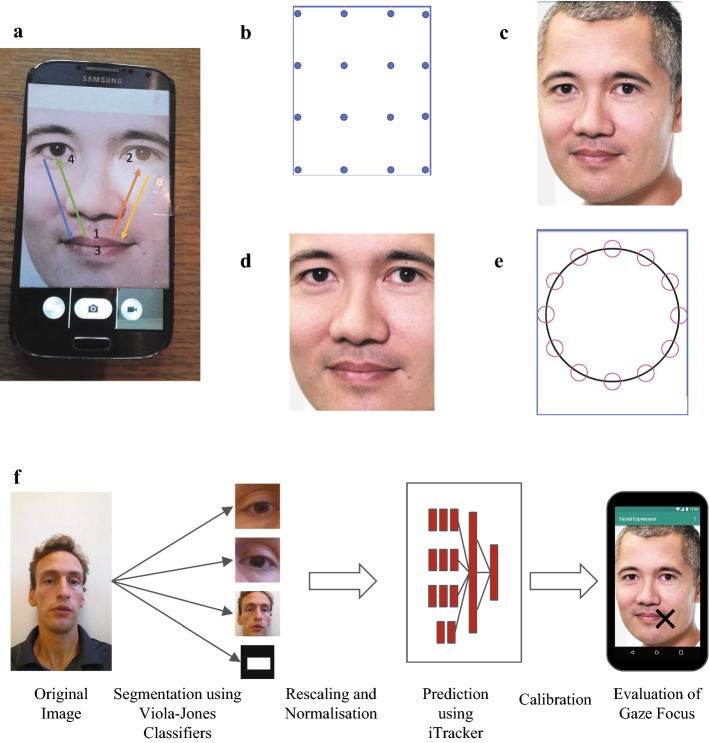
Study overview. We evaluated if iTracker [11] can provide a cheap, widely deployable method for tracking gaze behaviour using smartphones in ASD patients. **a** Example of the data collection set-up. We recorded subjects alternating between fixating on the eyes and the mouth of a face printout attached to the screen. Arrows indicate the sequence in which the different facial features were visited. Based on the so obtained videos, we evaluated how well iTracker can distinguish between the two gaze locations. **b**–**e** True gaze locations for each of the four tasks in our study. **b** Task 1: A 4x4 grid of points used for calibration. **c** Task 2: A face to test how accurately iTracker can separate gaze towards the eyes from gaze focussed on the mouth. **d** Task 3: Enlarged version of **c**, to test if separating eyes and mouth improves the ability to distinguish between gaze towards the eyes, and gaze towards the mouth. **e** Task 4: Subjects trace out a circle. **f** Outline of the data processing work flow: The obtained videos were split into frames, pre-processed, and gaze predictions obtained with iTracker. Predictions may be refined using a further calibration step

The aim of the present study was to prototype a smartphone-based gaze-monitoring framework, assess its accuracy on healthy volunteers, and identify key challenges to be overcome on the way to the clinic. The test set-up employed imitates that of, for example [12], in which the subject is shown the image of a face and one compares fixation time on the eyes to fixation time on the mouth. To estimate the gaze location, we used the convolutional neural network iTracker [11], which to the best of our knowledge has the best-reported performance on a smartphone in literature. The network was trained on the largest eye-tracking dataset to-date, consisting of around 1.2 million images of 1271 subjects recorded under home-use conditions. The authors report a mean Euclidean Error of 2.04 cm, with further reduction to 1.04 cm through a number of refinement steps (data augmentation, restriction to phone images, and calibration) [11]. We test the set-up on 17 healthy volunteers, and evaluate its accuracy for 2 different types of tasks: (i) separating between gaze towards the eyes and towards the mouth of a face, and (ii) resolving a more fine-grained gaze pattern (see Fig. 1 for an overview of our methods). In addition, we investigated how the accuracy is influenced by the distance between the eyes and the mouth, and how well accuracy can be improved through calibration. We compare two calibration methods: (i) a support vector regression (SVR)-based method proposed in [11], and (ii) a linear transformation-based method proposed by us. The SVR uses 128 features extracted from the final layer of the neural network to adjust the prediction, whereas the linear transformation merely translates points and rescales distances. We make our eye-tracking pipeline available online (https://github.com/ms234/iTrackerWrapper), and hope that our work will serve as a stepping stone to the creation of a tool which will help to improve the understanding and treatment of ASD in the future.

## Results

### iTracker captures patterns but its predictions are biased

**Fig. 2:**
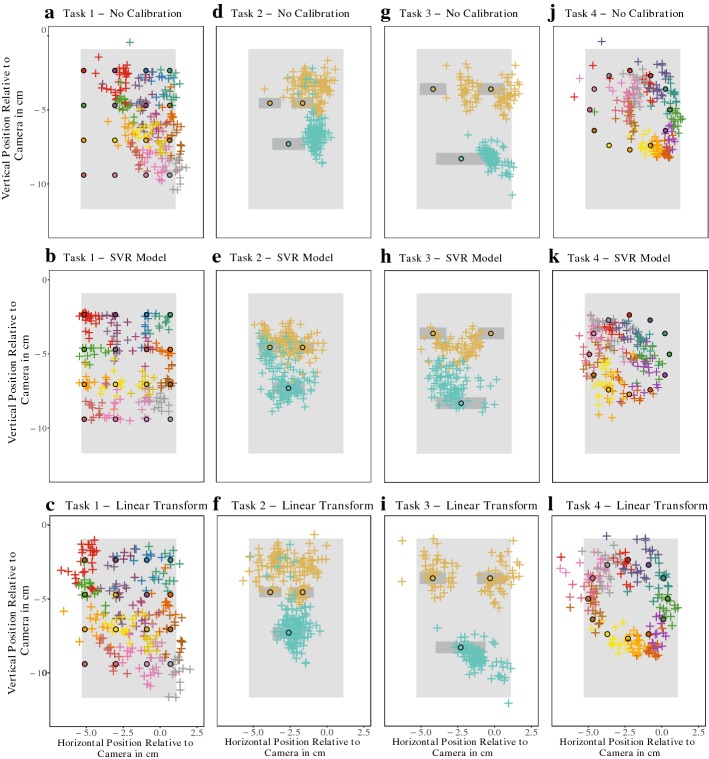
Results for one subject in our study (Subject 8). Crosses mark iTracker’s predictions. Points in matching colour indicate the true gaze locations for those predictions. Shaded areas represent the phone screen, and for Task 2 and 3 also the outline of the eyes and mouth. **a**–**c** Gaze estimates for Task 1. **d**–**f** Estimates for Task 2. **g**–**i** Gaze estimates for Task 3. **j**–**l** Estimates for Task 4. In the top row (**a**, **d**, **g**, **j**), the raw output of iTracker is shown. The middle and bottom row of the panel show these predictions corrected using either a SVR-based (**b**, **e**, **h**, **k**) or a linear transformation-based calibration method (**c**, **f**, **i**, **l**). Overall, iTracker manages to capture the true underlying pattern, although it appears shifted and scaled with respect to the reference (**a**, **d**, **g**, **j**). Calibration can rectify this, resulting in good overlap between true and estimated gaze positions (middle and bottom row; see also Fig. 3). Moreover, we find that the simple linear transformation performs better than the SVR-based method (compare middle and bottom row)

Figure 2 illustrates the results for one of the subjects in our study (Subject 8). Estimates for eyes and mouth cluster into easily distinguishable distinct patches (Fig. 2d, g). This separation is more pronounced in Task 3 than Task 2, where separate clusters for the left and right eye become visible. However, for neither task are the clusters centred on the eyes and mouth of the face on the screen. Instead they appear systematically shifted towards the right side of the screen. A similar pattern holds true for all subjects, with the magnitude and direction of the bias being conserved across different tasks for each subject but varying between subjects (for further examples see Additional file 1: Figures S1–S4).

**Fig. 3:**
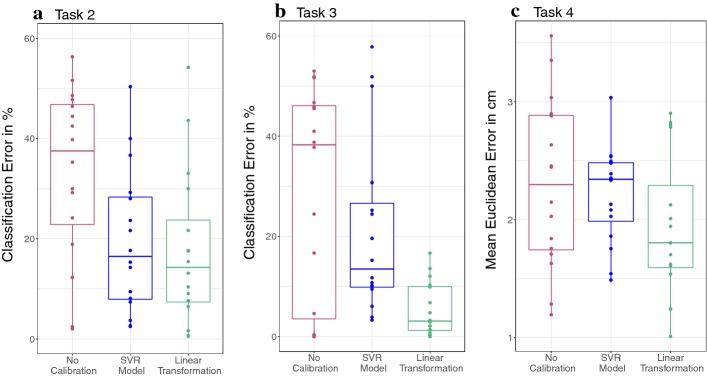
Quantification of iTracker’s Error without and with calibration. **a** Error in distinguishing between gaze towards eye and mouth of a face on screen (Task 2; Fig. 1c). Points were classified by which feature they were closest to. Shown is the proportion of wrongly assigned frames for each subject. Following calibration, both accuracy and variance improve. **b** Results for Task 3, which was similar to Task 2, but with an enlarged face in which eyes and mouth are further apart (Figure 1d). Performance appears more variable than for Task 2, but after post-processing with the linear calibration method very good accuracy and robustness is achieved. **c** Participants traced out the outline of a circle (Task 4; Fig. 1e). Shown is the mean Euclidean distance between the prediction and the true outline of the circle for each subject. Again calibration reduces variance and improves accuracy

In order to assess the accuracy with which the gaze location can be identified from the collected data, we classified estimates to belong to either the eye or mouth according to which they were closer to. The error of this classification is shown in Fig. 3. In Task 2, on average 33.23% of a subject’s frames are miss-classified (95% CI [24.08%, 42.39%]). In Task 3, we observe a reduction in the mean classification error to 28.65%, albeit the difference is not statistically significant (95% CI [17.23%, 40.08%]; Wilcoxon Signed-Rank Test, *V* = 93, *p* value = 0.21). In addition, there is large variation in accuracy between subjects, in particular for Task 3 (Fig. 3a, b).

### Calibration significantly improves robustness

While the performance of the SVR-based calibration method proposed in [11] reduces the error for Task 2 (Wilcoxon Signed-Rank Test: *V* = 110, *p* value = 0.03), it does not provide a statistically significant improvement for Task 3 (Wilcoxon Signed-Rank Test: *V* = 79, *p* value = 0.29; see also Fig. 3). Visual inspection of the gaze predictions suggests that this might be due to a tendency to cluster the points around the centre of the screen, thereby loosing structure previously visible in the data (see Fig. 2e, h, and k, and Additional file 1: Figures S1–S4).

Motivated by the observation that iTracker’s estimates appeared to be linearly shifted with respect to the reference points (e.g. Fig. 2b, g), we evaluated a calibration method based on a linear transformation. As we illustrate for one subject in Fig. 2f, i, the estimates now overlap well with the true gaze locations. Accordingly, compared to the unadjusted iTracker output, the mean classification error for Task 2 is reduced to 17.67% (95% CI [9.38%, 25.96%]; Wilcoxon Signed-Rank Test: *V* = 106, *p* value = 0.01). For Task 3, this is even reduced to only 5.44% (95% CI [2.56%, 8.31%]; Wilcoxon Signed-Rank Test: *V* = 77, *p* value < 0.01). As such, the linear transformation yields as good of, and for Task 3 an even better, reduction in error than the SVR-based method (Wilcoxon Signed-Rank Test for Task 2: *V* = 75, *p* value = 0.41; Wilcoxon Signed-Rank Test for Task 3: *V* = 130, *p* value < 0.01; see also Fig. 3).

Moreover, calibration also improves the robustness of classification. In particular for Task 3, the variance in accuracy between different subjects is significantly lower if calibration is applied (Fig. 3b; Levene Test: *F* value = 4.54, *df* = (1.46), *p* value = 0.03851). Whereas not statistically significant, reductions in variance are also noticeable for Task 2 (Fig. 3a; Levene Test: *F* value = 0.67, *df* = (1.46), *p* value = 0.42).

### iTracker can resolve fine-grained movement

While our main aim was to assess the feasibility of distinguishing between two facial landmarks, it was also of interest how well iTracker could resolve a more fine-grained temporal sequence (Task 4). Figure 2j illustrates for one subject that iTracker’s estimates capture the correct shape, and temporal sequence of the trajectory. They are also systematically shifted and contracted, but this can again be addressed with calibration (Fig. 2k, l). For further examples, see Additional file 1: Figures S1–S4.

We show the accuracy of the estimates as the Euclidean distance to the true gaze location in Fig. 3c. Without calibration, the mean Euclidean error is 2.30 cm (95% CI [1.92 cm, 2.69 cm]). Calibration with SVR yields a mean error of 2.21 cm (95% CI [1.99 cm, 2.43 cm]), although the reduction is not statistically significant (paired *T* test: *df* = 15, *t* = 0.52, *p* value = 0.61). In contrast, the linear transformation yields a statistically significant reduction in the average error to 1.93 cm (95% CI [1.61 cm, 2.26 cm]; paired *T* test: *df* = 15, *t* = 2.41, *p* value = 0.03).

## Discussion

Advancements in gaze-tracking technology for tablets and phones suggest that soon eye tracking could be performed routinely in an everyday setting. Such systems hold the potential for the development of novel, gaze-based digital biomarkers to monitor disabilities, such as ASD. In the present study, we investigated the feasibility of developing such a tool with the current state-of-the-art technology, iTracker, and evaluated the accuracy based on specific biomarker-relevant tests performed with healthy volunteers.

Overall, iTracker allows distinction between gaze towards the eyes and the mouth of a face shown on the screen. While distinction is possible for the smaller of the two faces used in this study (17.67% ± 8.29%), performance is significantly more robust when the distance between eyes and mouth is maximised (5.44% ± 2.88 %; Fig. 3a, b). We, thus, recommend to place the eyes and mouth as far apart as possible on the screen. In addition, we find that more fine-grained temporal sequences can be resolved with acceptable accuracy, as shown for Task 4 (Fig. 2). Our results thereby also independently confirm the error estimates for iTracker reported in [11] (2.04 cm; here: 2.30  ± 0.38 cm), and furthermore indicate that iTracker’s performance is relatively robust to the phone type used: iTracker was trained on iPhones whereas we used Android devices.

Moreover, in accordance with [11], we find that accuracy can be improved by post-processing the predictions with an error model trained on a calibration data set collected at the start of the experiment. However, we observed that while the SVR-based method proposed in [11] reduced the distance between true gaze location and the estimates, it tended to do so by clustering the predictions in the centre of the screen, loosing spatial structure previously visible (e.g. Fig. 2e, h, and k). As an alternative, we tested a simple linear error model to shift the estimates and rescale distances. Under the conditions of our experiment, this outperformed the SVR-based method and preserved the structure in the data better. We hypothesise that this is due to the small number of data points available for calibration (only 16 distinct locations), resulting in over-training of the SVR. In addition, the highly controlled circumstances of our experiments will have helped to reduce the error making a linear error model sufficient. It seems likely that in less-controlled environments (e.g. difficult lighting conditions, tremor when holding the phone), a non-linear model such as the SVR might be required. It would be important in the future to investigate this issue further and trial other statistical techniques, such as generalised linear models, to develop a method that can robustly correct for errors induced by differences in phone hold, lighting conditions, and visual appearance (e.g. skin colour). It might also prove beneficial to explicitly incorporate data such as gyroscope data to allow further personalisation.

Although we did not explore this question in detail, we noticed a strong dependence of iTracker’s performance on the distance between the screen and the user’s face (Additional file 1). In preliminary experiments, we found that the phone had to be held very close to the face for accurate and consistent performance (at most 20 cm). This distance is shorter than the distance most users would usually hold the phone at, and as such in a practical setting would require a test to ensure this distance is kept. We also noticed an influence of the phone angle on performance, although we were unable to clearly characterise the relationship. Given that the target population includes young children with challenging behaviour and cognitive disabilities, such sensitivity to the phone hold imposes serious restrictions on its use. Research to improve robustness should be a priority in developing this technology further.

To use iTracker, one has to accurately identify the face and the eyes in the image. Despite a long-lasting history of research in this area, a state-of-the-art available implementation failed surprisingly many times. We were only able to segment 74.7% of the frames. Similarly, Krafka et al. [11] who used the inbuilt iOS algorithm reported a 61% segmentation rate (only 1,490,959 of 2,445,604 had both face and eye detection [11]). Considering the large amount of potentially insightful data that are lost, this issue should be given further attention.

A great challenge in developing and benchmarking eye-tracking devices is to obtain accurately labelled validation data. In this study, we aimed to ensure accurate labelling of our data through manual validation. As a result, our data are likely biased towards easy images, since a human observer had to be able to identify the gaze location. Thus, the reported accuracies should be seen as best-case estimates. For further validation, it would be helpful to compare the performance of iTracker with that of a professional eye-tracking device. In addition, it could be interesting to repeat our experiment with other eye-tracking algorithms for phones, such as MPIIGaze [10], to compare their accuracies.

The aim of this study was to evaluate whether smartphone-based gaze monitoring is sufficiently accurate and robust to be employed in ASD research. This is the reason why we worked with healthy volunteers instead of patients. After the observed sensitivity to the phone hold is addressed, this study should be repeated with a cohort of ASD patients to investigate whether it can capture the differences in gaze patterns in practice. It would also be interesting to test if it can recapitulate the attraction towards non-social extraneous objects in visual scenes, or indeed the reduced time spent looking at the screen during the test, that has been found even more predictive of autism than aversion of direct eye contact [13]. If so, this might provide an alternative marker that could be monitored with a smartphone-based framework. Based on our analyses it seems plausible that such differences might be detectable, as the eyes and mouth in Task 2 were only 3 cm apart. Moreover, while we chose to focus our application on ASD, some of our results might be transferable to other mental disorders characterised by alterations in gaze behaviour. Tasks such as Task 4, for example, could be used to assess smooth pursuit dysfunction in Multiple Sclerosis [14, 15]. By presenting a prototype, and making our code publicly available, we hope to stimulate future research into these directions.

## Conclusions

We simulated a smartphone application in which participants are shown images of faces, and examined the accuracy with which iTracker [11] could distinguish between gaze fixations on eyes and mouth. We conclude that comparing gaze fixations towards the eyes and the mouth of a face shown on the screen is feasible with current technology. A calibration step will be required and care will have to be taken that the phone is held sufficiently close to the face. But, provided this is the case, we confirmed it is possible to obtain accurate estimates of the gaze position. Accuracy can be further improved by maximising the distance between eyes and mouth of the face shown on the screen. Future research should explore how iTracker performs under different recording conditions, to develop solutions to improve its robustness to the distance between the phone and the user and the angle at which the phone is held at, and explore the optimal calibration strategy. Assuming these concerns are addressed, we are confident that it will be soon possible to monitor gaze behaviour using smartphone-based applications.

## Methods

### Data collection

To benchmark iTracker’s accuracy, we carried out a proof-of-principle study on 17 healthy volunteers, in which we collected a set of front-camera images from phones for which the subject’s true gaze focus was known. The purpose of the study was solely to assess the accuracy of the software, with no medical implications. Eight of the subjects were based in Oxford, UK, and nine in Basel, Switzerland. All subjects were over 18 year old, and gave written consent to participate in the study. The phones used were a Samsung Galaxy S4 (8 subjects), and a Samsung Galaxy S7 (9 subjects). Participants were seated in front of a neutral background (white or grey wall), and were instructed to hold the phone at < 20 cm distance from their head at head height. Spectacle wearers were asked to take off their glasses. Participants carried out four tasks in which they traced out specific patterns on the screen with their eyes (Fig. 1a–e) while a video was taken. The patterns consisted of a set of way points printed on a piece of paper, which was attached to phone screen and was meant to simulate the smartphone application (Fig. 1a). Participants traced out the patterns by focussing on each way point for about 1s, after which they changed their focus to the next way point. Timing was enforced through use of a metronome. Subjects were allowed to trace out the pattern using their finger and follow their finger with their eyes. The four tasks consisted of:

- *Task 1* Calibration Grid: A 4 $\times$ 4 grid of points on the screen which was used for calibration (Fig. 1b).
- *Task 2* Original Face: Participants were shown the image of a face, and alternated their focus between the eyes and the mouth of the face, following the sequence: mouth, left eye, mouth, right eye (Fig. 1a, c). The pattern was repeated 5 times. Distance between eyes and mouth: 3.1 cm.
- *Task 3* Enlarged face: As Task 2, but with a digitally enlarged version of the same face (Fig. 1d). Distance between eyes and mouth: 4.3 cm.
- *Task 4* Circle: A circle on the screen consisting of 12 way points (Fig. 1e).

### Data processing and gaze prediction

Frames were extracted from the videos at 30fps and reviewed manually. Frames in which subjects blinked, or accidentally looked elsewhere on the screen were manually reallocated or excluded. The number of frames extracted for each task varies between subjects (see Additional file 1: Table S1). Next, the images were processed and gaze predictions were obtained with iTracker. The required crops of the face and eyes were extracted using the Viola–Jones detector in OpenCV [16], and rescaled and centred following the instructions in [11] and [17]. One subject (Subject 13) had to be excluded at this point from further analysis, as the face detection algorithm was unable to identify the face and eye regions. A significant number of frames from other subjects were also affected (see Additional file 1: Table S1). In total, we obtained 16,517 labelled images, distributed across 16 subjects, which we based our analysis on. Due to better performance in preliminary benchmarking tests (not shown), we chose the basic version of iTracker (“itracker_iter_92000.caffemodel” at [18]), instead of the also provided “$25\times$ train-augmented” version. Our pipeline is implemented in Python 2.7 [19], using scikit-video [20], opencv-python 3.1.0. [16], and Caffe 1.0 [21]. Our code is available at: https://github.com/ms234/iTrackerWrapper.

### Error metrics

Accuracy of automatic classification was evaluated with the following error metrics: For Tasks 2 and 3, we classified predictions according to which facial feature they were closest to (left, right eye, or mouth), as measured by Euclidean distance to the centre of that feature, and we report the proportion of misclassified frames per subject. Task 4 was mainly assessed visually. However, we also report the mean Euclidean distance between the predicted gaze focus and the true gaze focus for each subject (in centimetres).

### Calibration methods

We compared the improvements achieved by two calibration methods: (1) the SVR-based calibration method proposed in [11], and (2) a linear transformation-based method proposed by us. The methods were trained on the data collected in Task 1, and then applied to correct the predictions from Task 2–4. Accuracy was evaluated as before. The SVR-based method was implemented following [11], using the e1071 package in R [22]. The linear transformation was motivated by the observation that the gaze patterns captured by iTracker were shifted and stretched compared to the true patterns (see Fig. 2 for an example). Thus, we used a linear transformation to translate the predictions so that the centroid of the predictions coincided with the centroid of the true dots. In order to correct the length scale, we chose the transformation so that so that the variance in distance from the centroid is preserved between the set of predictions and the set of true gaze locations. It can be shown that the transformation which satisfies these requirements is given by,

$$\begin{aligned} \tilde{x} = a_x \hat{x} + b_x, \quad \text {and} \quad \tilde{y} = a_y \hat{y} + b_y, \end{aligned}$$

where $\left( \hat{x},\hat{y}\right)$ is the original prediction, $\left( \tilde{x},\tilde{y}\right)$ is the corrected prediction, and

$$\begin{aligned} a_x = \sqrt{\frac{\sigma ^2_x}{\hat{\sigma }^2_x}}, \quad a_y = \sqrt{\frac{\sigma ^2_y}{\hat{\sigma }^2_y}}, \quad b_x = \mu _x - a_x \hat{\mu }_x, \quad \text {and} \quad b_y = \mu _y - a_y \hat{\mu }_y. \end{aligned}$$

Here, $\mu _x$ and $\mu _y$ denote the mean, and $\sigma ^2_x$ and $\sigma ^2_y$ denote the variance of the x- and y-coordinate values of the grid points in Task 1. Similarly, $\hat{\mu }_x$ and $\hat{\mu }_y$ stand for the mean, and $\hat{\sigma }^2_x$ and $\hat{\sigma }^2_y$ stand for the variance in x- and y-coordinate values of the predicted positions.

### Statistical analyses

Performance was compared using paired t-tests, if the data followed a normal distribution (*p* value from Shapiro Wilk Test > 0.05 for both samples). Otherwise, we used Wilcoxon Signed-Rank Tests. To compare inter-subject variance in performance prior and post calibration, we used a Levene test for homoscedasticity. The post calibration sample for this test was obtained by pooling the results for both calibration methods. All statistical analyses were carried out in R version 3.4.0. [23].

## Additional file

**Additional file 1.** The Appendix contains gaze predictions for four further subjects from the study, and a table with a detailed overview of the results for each subject. Finally, we also present preliminary work on the inuence of the distance between the user and the phone on iTracker's accuracy.

## Abbreviations

ASD: autism spectrum disorders
SVR: support vector regression
