# Supplementary material for: Look me in the eye: evaluating the accuracy of smartphone-based eye tracking for potential application in autism spectrum disorder research
Source: Biomed Eng Online. 2019 May 3;18:51. doi: 10.1186/s12938-019-0670-1 (PMC6499948; doi:10.1186/s12938-019-0670-1)
Supplement: Supplementary file 1 — Additional file 1. The Appendix contains gaze predictions for four further subjects from the study, and a table with a detailed overview of the results for each subject. Finally, we also present preliminary work on the inuence of the distance between the user and the phone on iTracker's accuracy. [file 12938_2019_670_MOESM1_ESM.pdf]

Additional File

S1. Further Examples

In this section, we present gaze predictions for four further subjects. These illustrate two cases in which gaze prediction worked well (Fig. S1 and Fig. S2), and two cases in which our pipeline performed poorly (Fig. S3 and Fig. S4). The arrangement and labels follow the same scheme as Fig. 2.

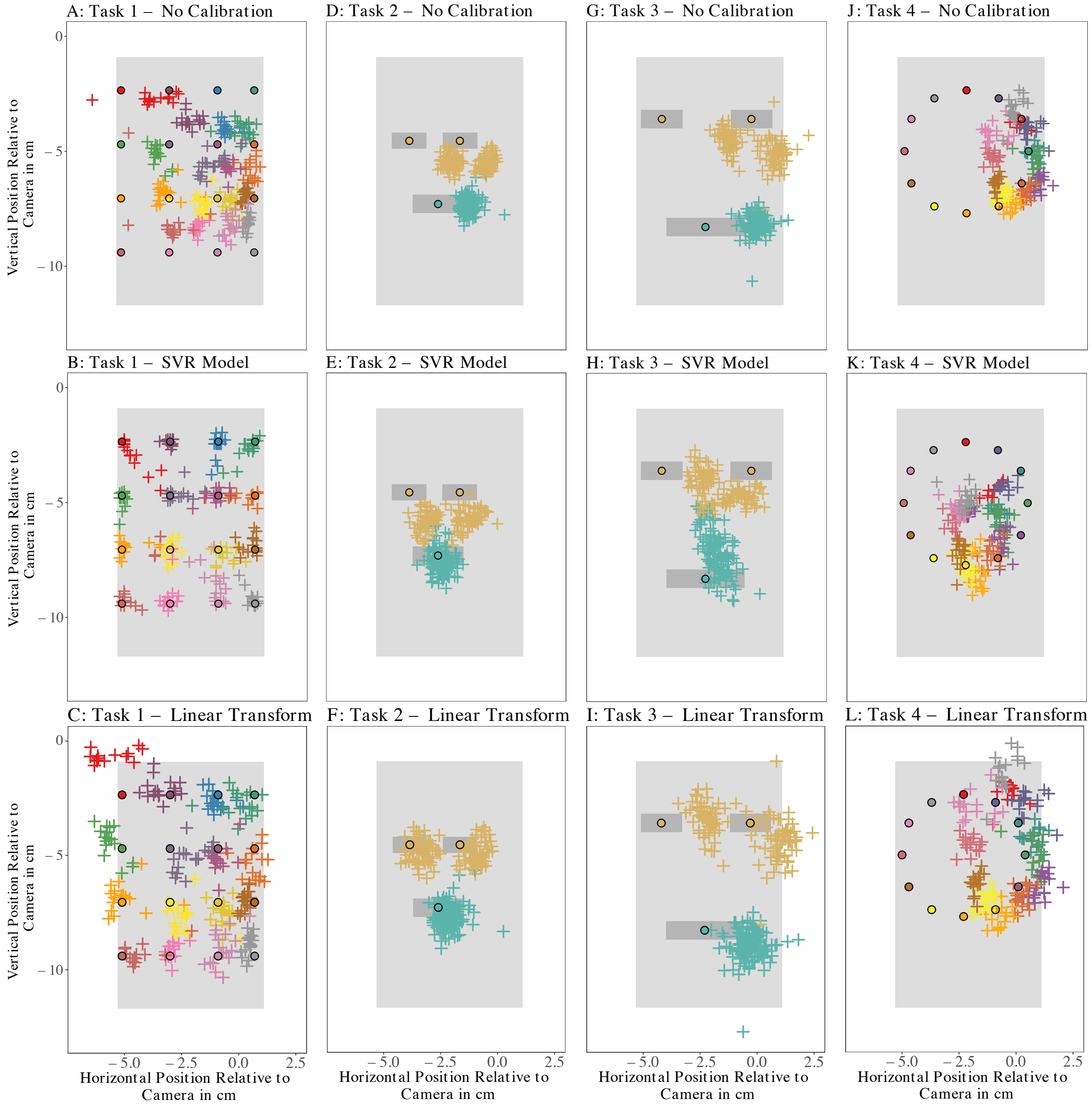

FIGURE S1. RESULTS FOR SUBJECT 3. LAYOUT AS IN FIG. 2. EYES AND MOUTH FRAMES ARE CLUSTERED INTO DISTINCT PATCHES, AND AFTER APPLICATION OF THE LINEAR TRANSFORMATION, OVERLAP WELL WITH THE POSITION OF THE EYES AND MOUTH ON THE IMAGE. THE CIRCLE IS LESS WELL RESOLVED.

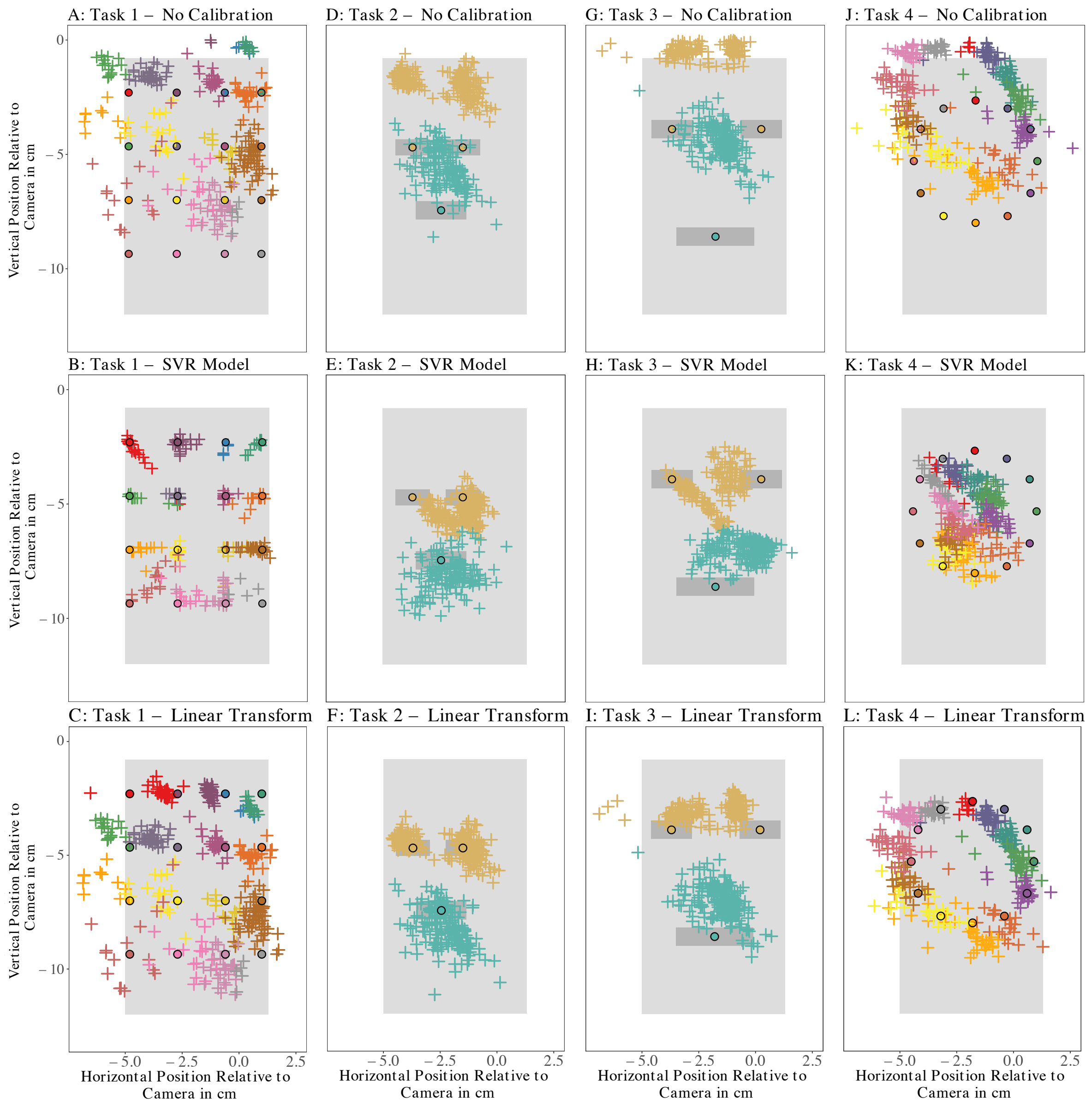

FIGURE S2. RESULTS FOR SUBJECT 18. LAYOUT AS IN FIG. 2. AGAIN, EYES AND MOUTH FRAMES ARE SEPARATED WELL AND ALIGNMENT IS CORRECTED BY THE LINEAR TRANSFORMATION. IN THIS CASE ALSO THE CIRCLE IS WELL RESOLVED.

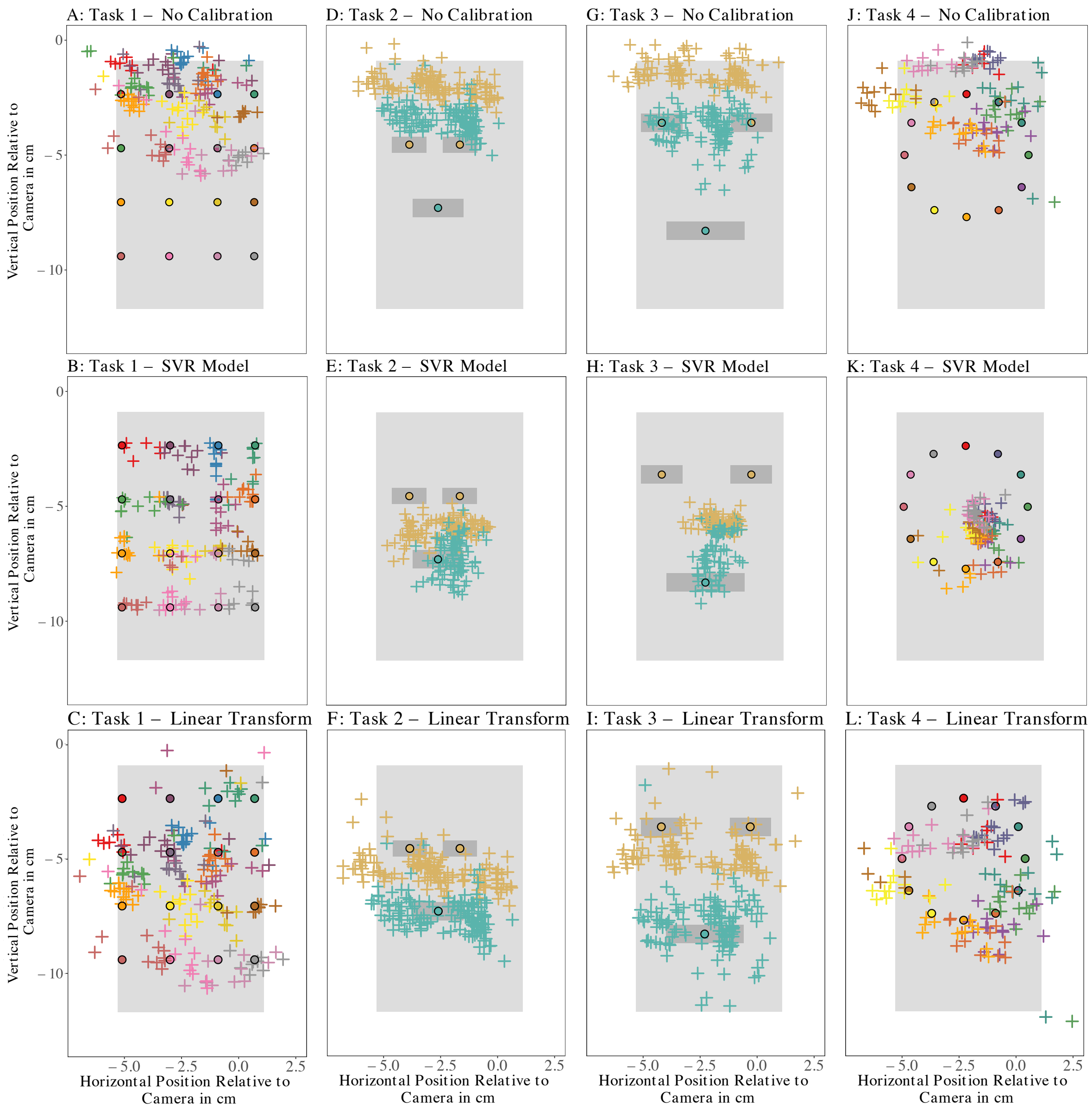

FIGURE S3. RESULTS FOR SUBJECT 6. LAYOUT AS IN FIG. 2. WHILST EYE AND MOUTH FRAMES SEPARATE, THE DISTINCTION IS LESS CLEAR THAN IN PREVIOUS EXAMPLES (FIG. 2, FIG. S1, AND FIG. S2). POSSIBLY THE PARTICIPANT SLIPPED, AS THERE APPEAR TO BE TWO DIFFERENT FOCI ON EACH EYE AND THE MOUTH, SUGGESTING THE PARTICIPANT MIGHT HAVE CHANGED THE TARGET THEY FOCUSSED ON (E.G. FOCUS ON EYE LID INSTEAD OF PUPIL; BEST SEEN IN I)). THE SVR CALIBRATION METHOD CLUSTERS POINTS IN THE CENTRE, AND DESTROYS PREVIOUSLY EXISTING STRUCTURE (E, H, K).

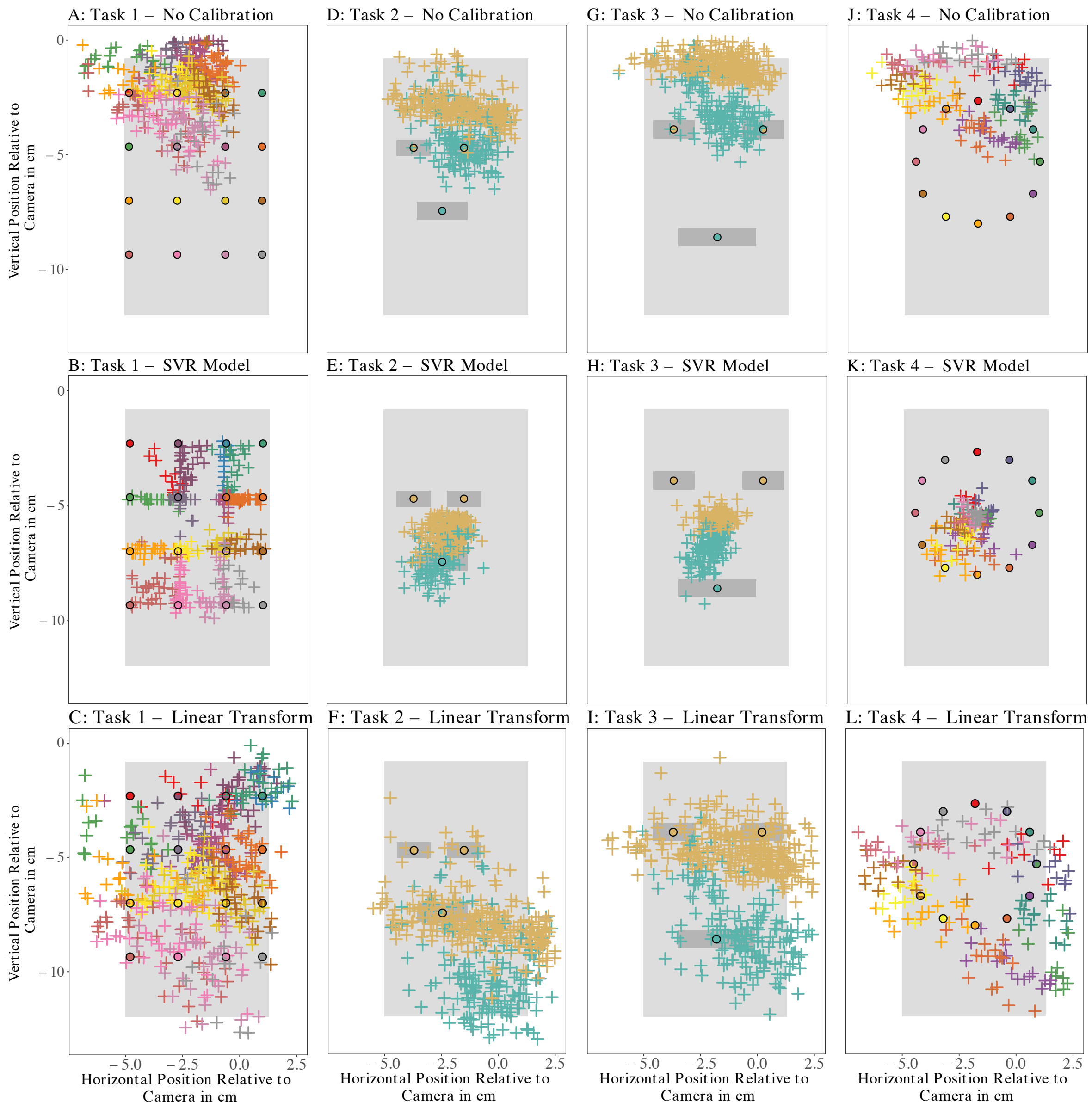

FIGURE S4. RESULTS FOR SUBJECT 14. LAYOUT AS IN FIG. 2. EYE AND MOUTH FRAMES ARE SEPARATED, BUT THERE IS LARGE VARIABILITY IN THE PREDICTIONS (E.G. RIGHT AND LEFT EYE CAN NOT BE DISTINGUISHED). THIS PARTICIPANT HAS THE LARGEST AVERAGE CLASSIFICATION ERROR ACROSS TASK 3 AND TASK 4 (SEE ALSO TABLE S1).

S2. Table Summarising the Benchmarking Data Set and Classification Accuracy for Each Subject

TABLE S1. #FRAMES REFERS TO THE NUMBER OF IMAGES COLLECTED FOR THIS SUBJECT (AFTER MANUAL REVIEW). #SEGMENTED INDICATES THE NUMBER OF THESE IMAGES, FOR WHICH WE WERE ABLE TO SEGMENT THE FACE AND EYES. THESE WERE THE IMAGES OUR ERROR ASSESSMENT IS BASED ON. ERROR REFERS TO CLASSIFICATION ERROR (FOR TASK 2 AND 3), AND EUCLIDEAN ERROR (TASK 4), RESPECTIVELY.

Subject	Phone	Task 1		Task 2			Task 3			Task 4		
		#Frames	#Segmented	#Frames	#Segmented	Error	#Frames	#Segmented	Error	#Frames	#Segmented	Error
1	Galaxy S4	366	366	248	247	2%	269	268	0%	261	246	1.7cm
2	Galaxy S4	253	253	181	180	47.8%	213	184	24.5%	200	200	1.28 cm
3	Galaxy S4	270	270	353	245	2.4%	289	274	0.4%	197	197	2.45 cm
4	Galaxy S4	281	270	163	162	44.4%	183	182	46.7%	198	198	2.15 cm
5	Galaxy S4	253	253	151	149	24.2%	241	240	4.6%	225	225	1.84 cm
6	Galaxy S4	187	187	316	279	51.6%	239	238	51.7%	145	132	3.03 cm
7	Galaxy S4	217	217	300	261	56.3%	486	486	45.9%	197	197	2.45 cm
8	Galaxy S4	225	225	255	254	18.9%	186	186	0%	194	194	1.76 cm
10	Galaxy S7	150	139	921	564	46.5%	497	205	41%	248	248	3.35 cm
11	Galaxy S7	156	130	472	472	12.3%	513	491	0.2%	277	268	1.19 cm
12	Galaxy S7	167	64	369	282	48.6%	275	241	51.9%	400	400	2.88 cm
13	Galaxy S7	160	0	650	0	NA	480	0	NA	187	0	NA
14	Galaxy S7	503	503	465	465	39.8%	503	503	37.8%	183	183	3.56 cm
15	Galaxy S7	249	126	1032	34	35.3%	464	464	38.8%	250	248	2.03 cm
16	Galaxy S7	234	231	1173	20	30%	603	6	16.7%	121	110	1.63 cm
17	Galaxy S7	283	275	320	320	42.5%	290	290	45.5%	301	300	2.63 cm
18	Galaxy S7	342	342	380	380	29.2%	402	402	53%	346	346	2.9 cm

S3. Preliminary Results on the Relationship between iTracker's Accuracy and the Distance of the User to the Phone Screen

During our study, we noticed a dependence of iTracker's performance on the distance between the user and the phone. To investigate this in more detail, we compared the error in iTracker's estimates for the same task carried out at three different distances to the screen (20 cm, 40 cm, 60 cm) for one subject. The task consisted of subsequently focusing on each of 9 equally-spaced grid points which had been marked on the screen, and taking a picture. This was repeated five times at each distance. To fix the distance and phone hold, the phone was mounted in a car phone hold. The phone used was a Samsung Galaxy S4. The images were subsequently processed as described in Section 3.2..

Fig. S5 shows the results of our experiment. As can be seen, the error consistently decreases, as the user comes closer to the screen (ANOVA, F-value = 8.28, p-value = 0.0047). Best results are achieved when the phone is 20 cm from the face. However, as Fig. S5B illustrates, even in this case the predictions still cluster around the centre of the screen. Thus, for our experiment we asked participants to hold the phone at 15-20cm from their face. As this is closer than most users naturally hold a phone at, this issue should be investigated in more detail to make iTracker more robust in a general use case.

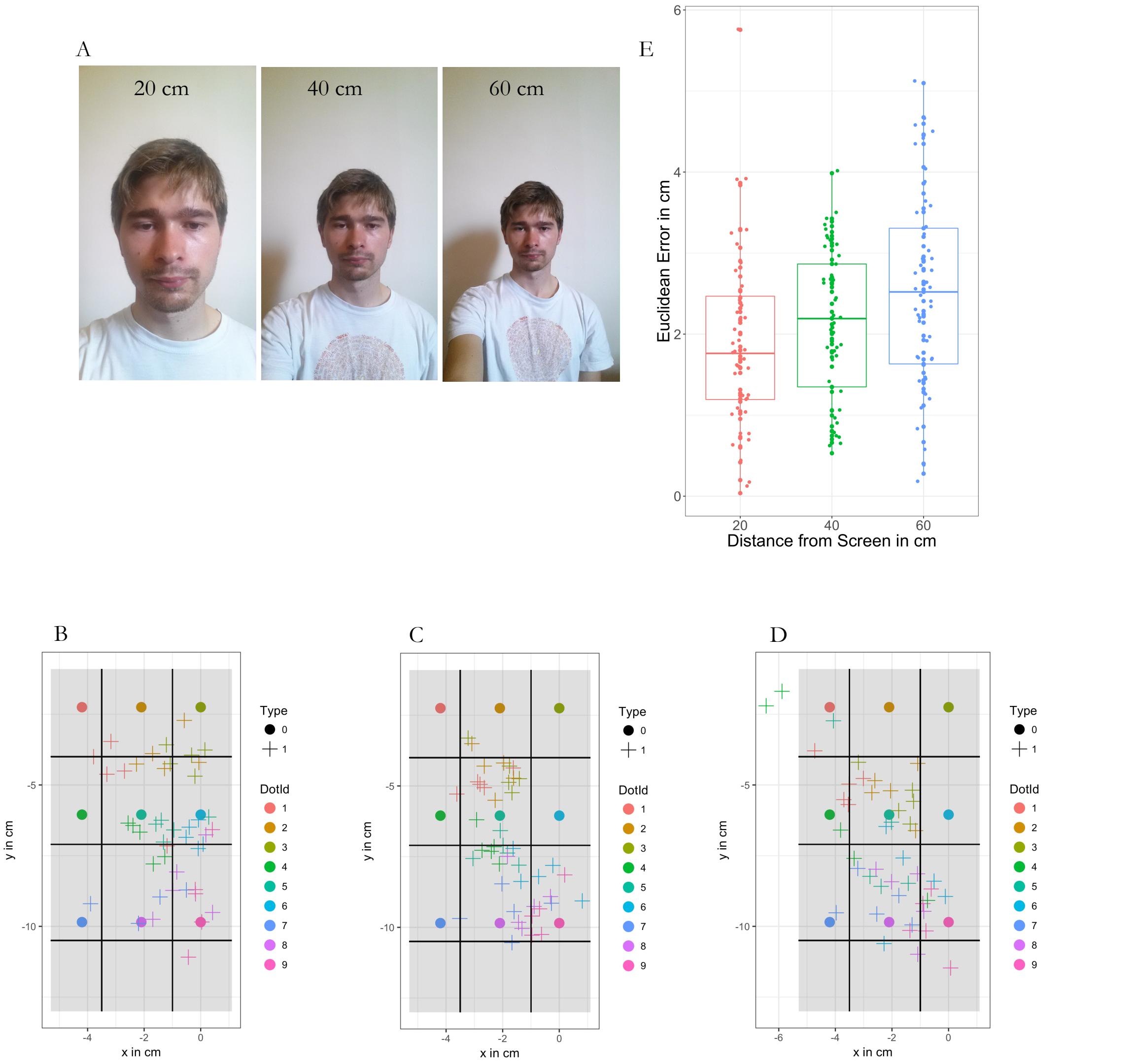

FIGURE S5. INFLUENCE OF THE DISTANCE OF THE USER FROM THE SCREEN ON GAZE MEASUREMENT ACCURACY. ACCURACY WAS TESTED AT 3 DISTANCES, KEEPING THE HEAD FOCUSED IN THE CENTRE OF THE IMAGE (20 CM, 40 CM, AND 60 CM). A) EXAMPLE IMAGES AT EACH DISTANCE. B)-D) GAZE LOCATIONS PREDICTED BY ITRACKER FOR DISTANCE 20CM (B), 40CM (C), AND 60CM (D) (CROSSES: PREDICTIONS; FILLED CIRCLES: TRUE GAZE LOCATION; TRUE LOCATION, AND CORRESPONDING PREDICTIONS ARE COLOURED IN THE SAME COLOUR). E) EUCLIDEAN ERROR FOR EACH IMAGE AT EACH OF THE THREE DISTANCES. THE RESULTS SHOW THAT THE CLOSER THE FACE IS TO THE SCREEN, THE BETTER THE ACCURACY OF THE PREDICTIONS. HOWEVER, EVEN AT 20CM VISUAL INSPECTION (B) SHOWS THAT THERE IS STILL A TENDENCY OF PREDICTIONS TO CLUSTER IN THE CENTRE. THUS, FOR OUR STUDY WE USED A DISTANCE OF 15-20CM.
